# Burden of scabies in displacement settings: A systematic review and meta-analysis among forcibly displaced populations

**DOI:** 10.1371/journal.pntd.0013853

**Published:** 2025-12-23

**Authors:** Gelila Yitageasu, Angwach Abrham Asnake, Alemayehu Kasu Gebrehana, Bizunesh Fantahun Kase, Hiwot Altaye Asebe, Mitkie Tigabie, Lidetu Demoze

**Affiliations:** 1 Department of Environmental and Occupational Health and Safety, Institute of Public Health, College of Medicine and Health Sciences, University of Gondar, Gondar, Ethiopia; 2 Department of Epidemiology and Biostatistics, School of Public Health, College of Health Sciences and Medicine, Wolaita Sodo University, Wolaita Sodo, Ethiopia; 3 Department of Midwifery, College of Health Sciences, Salale University, Fitche, Ethiopia; 4 Department of Public Health, College of Health Sciences, Mattu University, Mattu, Ethiopia; 5 Department of Public Health, College of Medicine and Health Sciences, Samara University, Samara, Ethiopia; 6 Department of Medical Microbiology, School of Biomedical and Laboratory Science, College of Medicine and Health Sciences, University of Gondar, Gondar, Ethiopia; Hebrew University Hadassah Medical School, ISRAEL

## Abstract

**Background:**

By the end of 2024, an estimated 123.2 million people were forcibly displaced due to conflict and persecution, with 80% residing in low- and middle-income countries. Scabies, a neglected tropical disease affecting around 200 million people globally and causing over 455 million new cases annually, disproportionately affects displaced populations due to overcrowding, poor hygiene, and limited healthcare access. However, global evidence on its prevalence among these groups remains inconsistent. This study aimed to estimate the global pooled prevalence of scabies among forcibly displaced populations.

**Methods:**

Systematic searches were conducted in PubMed, Epistemonikos, ScienceDirect, Scopus, and Embase for studies published up to May 28, 2025, supplemented by hand-searches using Google and Google Scholar to capture any additional relevant studies. Data were extracted using Microsoft Excel 2021 and analyzed with STATA version 17. We conducted a systematic review and meta-analysis using the Preferred Reporting Items for Systematic Reviews and Meta-Analyses (PRISMA) guidelines. Only studies that met the predefined inclusion criteria were included. The Newcastle-Ottawa Scale assessed study quality. A random-effects model was used to calculate pooled prevalence. Heterogeneity was measured using the Galbraith plot and the I^2^ statistic, while publication bias with funnel plots and Egger’s regression test. The protocol was registered with PROSPERO (CRD420251067217).

**Results:**

Fourteen studies with 76,664 participants were included. The global pooled prevalence of scabies was 15% (I^2^ = 99.87%). Prevalence varies by region, study setting, population group, and data collection method. Prevalence was highest in Asia (25%), followed by Europe (13%) and Africa (7%). Refugees showed the highest prevalence (23%), compared to IDPs (13%), asylum seekers (2%), and refugee-asylum seekers (2%). Among population groups, prevalence was highest in combined adult–child populations (66%), patients (24%), and school-aged children (15%), women and children under 15 years (6%), the general population (2%), and unaccompanied minors (1%). Studies using both clinical exams and Questionnaires reported a higher prevalence (27%) than those using clinical exams alone (14%) and interviews or physical examination only (3% and 2%, respectively).

**Conclusion:**

Scabies poses a significant health (15%) burden among forcibly displaced populations. Integrated interventions, including screening, treatment, WASH programs, and improved surveillance, are urgently needed to reduce transmission and improve health outcomes in displacement settings.

## Author summary

Worldwide, more than 120 million people have been forcibly displaced due to conflict and persecution, many of whom live in overcrowded camps or temporary shelters with limited access to water, sanitation, and healthcare. These challenging conditions increase the risk of scabies, a neglected tropical disease that causes intense itching, skin irritation, and secondary infections. Although scabies is widely recognized as a problem in displacement settings, its overall burden among forcibly displaced populations has not been well understood. In this systematic review and meta-analysis, we analyzed data from 14 studies including more than 76,000 participants living in various displacement contexts such as refugee camps, detention centers, and internally displaced persons (IDP) settlements. We found that scabies affects approximately 15% of forcibly displaced people globally, with the highest prevalence observed in Asia and among refugee populations. Prevalence also differed by age group, setting, and the type of diagnostic method used. Our findings demonstrate that scabies remains a substantial but often overlooked health issue in displacement settings. Strengthening screening, timely treatment, water and sanitation (WASH) interventions, and disease surveillance is crucial to reduce transmission and improve the well-being of affected populations.

## Introduction

Globally, the number of forcibly displaced individuals has been increasing at an alarming rate [1]. According to reports from the United Nations High Commissioner for Refugees (UNHCR), 108.4 million people were forcibly displaced by the end of 2022, rising to 123.2 million by the end of 2024, due to factors such as persecution, conflict, violence, human rights violations, or events that severely disrupt public order [2,3]. The term “forcibly displaced people” encompasses three primary groups: internally displaced persons (IDPs), refugees, and asylum seekers. Refugees are individuals who have fled their country of origin due to a well-founded fear of persecution and are unwilling or unable to return. IDPs are those who are forced to leave their homes for similar reasons as refugees but remain within their country’s borders [4,5]. Asylum seekers are individuals who have applied for refugee status and are awaiting a decision [3]. Armed conflict remains the leading driver of displacement, particularly among refugee populations, often resulting in the establishment of overcrowded and under-resourced settlements [6]. These conditions significantly increase the risk of communicable skin diseases, such as scabies, among displaced populations.

Scabies is a highly contagious skin infestation caused by the ectoparasite *Sarcoptes scabiei* var. *hominis*, and is primarily transmitted through prolonged skin-to-skin contact [7]. It is one of the most prevalent dermatological conditions, constituting a significant portion of skin diseases in developing nations [8]. Prevalence estimates vary significantly by region, ranging from 0.2% to 71.4%, with the highest rates reported in parts of the Pacific and Latin America, particularly among children [9,10]. In the Global Burden of Disease (GBD) 2015 study, scabies accounted for 0.21% of all Disability-Adjusted Life Years (DALYs), underscoring its global public health impact [11]. According to the World Health Organization (WHO) report globally, it is estimated to affect more than 200 million people at any time and more than 400 million people cumulatively every year [12]. Geographically, scabies infections are more common in locations with tropical climates, impoverished nations, and inadequate access to water [13]. The disease is often endemic in crowded living environments such as refugee camps, detention centers, and informal settlement settings.

Scabies imposes a substantial burden on healthcare systems and causes significant physical and psychological distress among affected individuals. Clinically, it is characterized by intense pruritus, often worsening at night, and inflammatory skin lesions typically found in the interdigital spaces, abdomen, and inguinal areas. Without timely diagnosis and treatment, the condition may lead to secondary bacterial infections, resulting in complications such as cellulitis, abscesses, impetigo, septicemia, glomerulonephritis, and, in severe cases, renal failure and rheumatic heart disease [14–16]. Chronic itching may also cause marked psychological and behavioral disturbances [16]. Additionally, the social stigma associated with visible skin conditions may deter individuals from seeking care, further perpetuating transmission and morbidity.

Various socioeconomic and environmental factors contribute to the occurrence and transmission of scabies. Individual characteristics such as age, sex, occupation, and household size, along with low education levels and poor hygiene practices, are associated with increased risk [17–21]. Prolonged skin contact and shared materials use (clothes, beds, and pillows) facilitate the spread of the mite [14,22]. According to Médecins Sans Frontières (MSF), overcrowded shelters, limited access to clean water, and frequent close contact in displacement settings create ideal conditions for rapid mite spread [23]. Poor sanitation infrastructure in IDP and refugee camps further heightens the risk of scabies and other communicable diseases [24]. Recurrent displacement due to armed conflict or environmental disasters often results in unstable shelter, restricted access to healthcare, and inadequate hygiene conditions that amplify disease vulnerability, especially among children and the elderly.

Despite the well-documented burden of scabies in overcrowded and resource-limited settings, no comprehensive systematic review or meta-analysis has synthesized the global prevalence of scabies specifically among forcibly displaced populations. To address this gap, the present study aims to estimate the pooled global prevalence of scabies among refugees, internally displaced persons, and asylum seekers through a systematic review and meta-analysis. By incorporating studies from multiple geographic regions, this review provides a consolidated understanding of both the magnitude and regional variation of scabies burden in humanitarian settings. The results will generate evidence to inform the design and implementation of targeted prevention and control strategies, guide health policy, and support the equitable allocation of resources for these high-risk populations.

## Methods

### Protocol and registration

This systematic review and meta-analysis were registered on PROSPERO with registration (CRD420251067217).

### Search strategies

This systematic review was conducted and reported following the Preferred Reporting Items for Systematic Reviews and Meta-Analyses (PRISMA **2020**) guidelines and checklist guidelines [25,26] (S1 File). A comprehensive literature search was conducted to identify all relevant peer-reviewed studies published up to May 28, 2025, using major electronic databases, including Medline/PubMed, Epistemonikos, ScienceDirect, Embase, and Scopus. These databases were selected to ensure comprehensive coverage of the existing published literature on the topic. To capture any potentially relevant studies not indexed in these databases, supplementary searches were performed using Google and Google Scholar Advanced Search. All Google Scholar results were exported to EndNote and screened in their entirety, rather than limiting to the first 200–300 hits, to ensure comprehensive inclusion of relevant studies. Google and Google Scholar were not included in the formal systematic search due to limitations in reproducibility (S2 File). The search strategy employed both Medical Subject Headings (MeSH) and entry terms, incorporating keywords such as *“*prevalence*”* AND “scabies” OR *“Sarcoptes scabiei infestation”* OR “scabies infestation” OR “human scabies” OR “scabies mite infestation” AND *“*internally displaced people” OR “refugee camp population” OR “refugee camp people*” OR “forcibly displaced people*” OR “conflict affected people*” OR “forcibly migrated people*” OR “asylum seekers” OR “armed conflict affected people*” OR “migrated people*” OR “migration camp*”. These terms were used both independently and in combination using Boolean operators (“AND” and “OR”) to refine the search and maximize the retrieval of relevant articles.

### Eligibility criteria

#### Inclusion criteria.

- **Language of Publication**: Only studies published in English were included, as English is the primary language of international scientific communication, most relevant studies were anticipated to be in English, and translation resources were not available.
- **Outcome of interest**: Studies were eligible if they reported the prevalence of scabies as a primary or secondary outcome among the target population.
- Study Population: The review focused on forcibly displaced populations, including internally displaced persons (IDPs), refugees, and asylum seekers.
- **Geographic Scope**: Studies conducted in any country or region globally were considered for inclusion.
- **Publication Date and year**: All relevant studies published up to May 28, 2025, were considered eligible.
- **Study Design**: Observational studies, including cross-sectional, case-control, and cohort designs, that reported the prevalence of scabies in the target population were eligible for inclusion.
- **Publication Status**: Only peer-reviewed articles published in scientific journals were included to ensure methodological quality and reliability of findings.

#### Exclusion criteria.

Studies were excluded if they did not report the prevalence of scabies among forcibly displaced populations or if full-text articles were unavailable despite at least three attempts to contact the corresponding authors. Additionally, non-original research formats, including systematic reviews, qualitative studies, letters, conference abstracts, short communications, commentaries, and case reports, were excluded. Conference abstracts and non-peer-reviewed sources were omitted to ensure the inclusion of high-quality studies with complete methodological details and results. These sources often lack sufficient information for critical appraisal and may not have undergone rigorous peer review, potentially compromising data accuracy and reliability.

### Outcome of interest

The primary outcome of this systematic review and meta-analysis was the pooled prevalence of scabies among forcibly displaced populations worldwide. Prevalence was defined as the proportion of individuals diagnosed with scabies divided by the total number of individuals assessed, multiplied by 100 to express the result as a percentage. Studies employed a variety of diagnostic approaches, including clinical examination alone, clinical examination combined with Questionnaires, questionnaire- or interview-based methods, and physical examination alone.

Variation in diagnostic methods may have introduced misclassification bias, as some approaches may under- or over-detect scabies cases. Subgroup analyses by diagnostic approach were conducted to address this bias. A summary of the diagnostic methods used in each included study is provided in Table 1 to improve transparency and facilitate interpretation of pooled prevalence estimates.

**Table 1:** Diagnostic methods used in included studies of scabies among forcibly displaced populations.

Author (s) and Publication Year	Study location and Country	Data collection tool	Diagnostic approach
Lafta R., et.al, 2016 [27]	Kirkuk, Baghdad, and Karbala, Iraq	Semi-structured questions	Questionnaire-Based/Interview Methods
Theuring S., et.al, 2016 [28]	Berlin, Germany	Questionnaires, medical history, and symptoms	Questionnaire-Based/Interview Methods
Ismael AF and El-Gilany A. 2015 [29]	Southern Chad, Chad	Clinical examination	Clinical examination
Rasul MM., et.al, 2019 [1]	Kaga Bandoro district, Central African Republic	Questionnaire-based interviews and diagnosis were made on clinical evaluation and laboratory methods.	Clinical Examination Combined with Questionnaires
Rahman MS., et.al, 2024 [23]	Teknaf/Ukhia Cox’s Bazar, Bangladesh	Clinical examination and Semi-structured questions	Clinical Examination Combined with Questionnaires
Abdullah AM., et.al, 2020 [30]	Duhok Refugees’ Camps, Iraq	clinical features of the patients and sometimes microscopic identification, or by using a dermatoscope	Clinical Examination Combined with Questionnaires
Zinszer K, and Abuzerr S., 2024 [31]	Gaza, Palestine	Structured questions	Questionnaire-Based/Interview Methods
Kortas AZ., et.al, 2017 [32]	Germany	Physical examination	Physical Examination Only
Wollina U., et.al, 2016 [33]	Dresden, Germany	Clinical examination	Clinical examination
Di Meco E., et.al, 2018 [34]	Lampedusa and Trapani-Milo, Italian	Clinical examination	Clinical examination
Alberfkani MI, and Mero WM, 2020 [35]	Cham Mishko Camp, Zakho City, Iraq	Physical examination and microscopic examination	Clinical Examination Combined with Questionnaires
Ibrahim AM., et.al, 2025 [36]	Qoloji IDP in Babile district, Somalia	Clinical examination and structured questions	Clinical Examination Combined with Questionnaires
Alemu T., et.al, 2022 [37]	Ranch collective site, Chagni town, Ethiopia	Clinical examination	Clinical examination
Alberer M., et.al, 2018 [38]	Munich, Germany	Clinical examination	Clinical examination

### Study selection, data extraction, and management

EndNote reference management software, version 20.5 (Thomson Reuters, Philadelphia, PA, USA) [39] was used to organize search results, remove duplicates, and exclude irrelevant titles and abstracts. Two reviewers (G.Y. and L.D.) independently screened titles, abstracts, and full-text articles based on predefined inclusion and exclusion criteria. The selected studies were then cross-checked by three additional reviewers (A.K.G., H.A.A., and A.A.A.), with any disagreements resolved through discussion and input from M.T. and B.F.K. as needed to reach consensus. Data extraction was performed independently by G.Y. and L.D. using a standardized spreadsheet, and discrepancies were resolved with consultation from the other team members. Extracted variables included author, year of publication, study period, study design, country, continent, study setting, target population, data collection method, sample size, response rate, number of scabies cases, and prevalence. To ensure methodological rigor, we conducted sensitivity and subgroup analyses, assessed publication bias, addressed missing data where possible, and adhered to standardized data extraction procedures.

### Risk of bias assessment

A full-text review was conducted for all eligible studies before inclusion in the final meta-analysis. The Newcastle-Ottawa Scale (NOS), adapted for both cross-sectional and case-control studies [6], was used to assess the methodological quality of the included studies. For cross-sectional studies, the maximum possible score was 10, while for case-control studies it was 9. Cross-sectional studies were categorized as follows: scores of 9–10 indicated very good quality, 7–8 indicated good quality, 5–6 were considered satisfactory, and 0–4 were considered unsatisfactory. As there is no universally accepted classification for case-control studies, we reported their quality scores on a 0–9 scale without categorization. For cross-sectional studies, quality assessment criteria included sample representativeness, sample size, response rate, ascertainment of exposure, comparability of subjects, control of confounders, appropriateness of statistical analysis, and outcome assessment. For case-control studies, assessment included the definition and representativeness of cases, selection and definition of controls, comparability between groups, exposure assessment methods, and nonresponse rate. Two reviewers (G.Y. and L.D.) independently assessed the methodological quality of the studies (S3 File). Disagreement between reviewers during the quality evaluation was resolved through discussion.

### Data processing and analysis

All extracted data were initially organized in Microsoft Excel 2021 and subsequently exported to STATA version 17 for statistical analysis (S4 File). To assess heterogeneity across studies, the I^2^ statistic was calculated, representing the percentage of total variation attributable to heterogeneity rather than chance. Values of 25%, 50%, and 75% were interpreted as low, moderate, and high heterogeneity, respectively [40]. In this review, substantial heterogeneity was observed (I^2^ = 99.87%, *p* < 0.001), indicating considerable variability across studies. Given the high level of heterogeneity, a random-effects meta-analysis model was employed using DerSimonian and Laird weighting to estimate the pooled prevalence of scabies and associated odds ratios, along with 95% confidence intervals [41]. This approach accounts for between-study variability, which is expected in studies conducted across diverse displacement settings and populations.

Subgroup analyses were pre-specified in the PROSPERO protocol as part of the planned strategy to investigate potential sources of heterogeneity. Based on the characteristics of the included studies, subgroup analyses were conducted based on study continent, subcontinent, data collection tool, publication year, study population and study setting (e.g., refugees, IDPs, or asylum seekers). Furthermore, a sensitivity analysis was performed to identify the impact of individual studies (extreme outliers) on the pooled estimate. In addition, sensitivity analysis was performed using a leave-one-out approach, where each study was excluded sequentially to assess its impact on the overall pooled estimate [42]. This allowed us to evaluate the robustness and influence of individual studies on the final result. To assess publication bias, both visual and statistical methods were used. A funnel plot was generated, and Egger’s regression test was performed, with statistical significance set at *p* < 0.05 [43]. Where asymmetry suggested potential bias, trim and fill analysis was conducted to estimate the number of potentially missing studies and adjust the pooled effect accordingly [44]. All analyses were interpreted in light of the study context, with careful consideration of heterogeneity, data quality, and methodological variability across included studies.

## Results

### PRISMA-based study identification and inclusion flow

**Fig 1:**
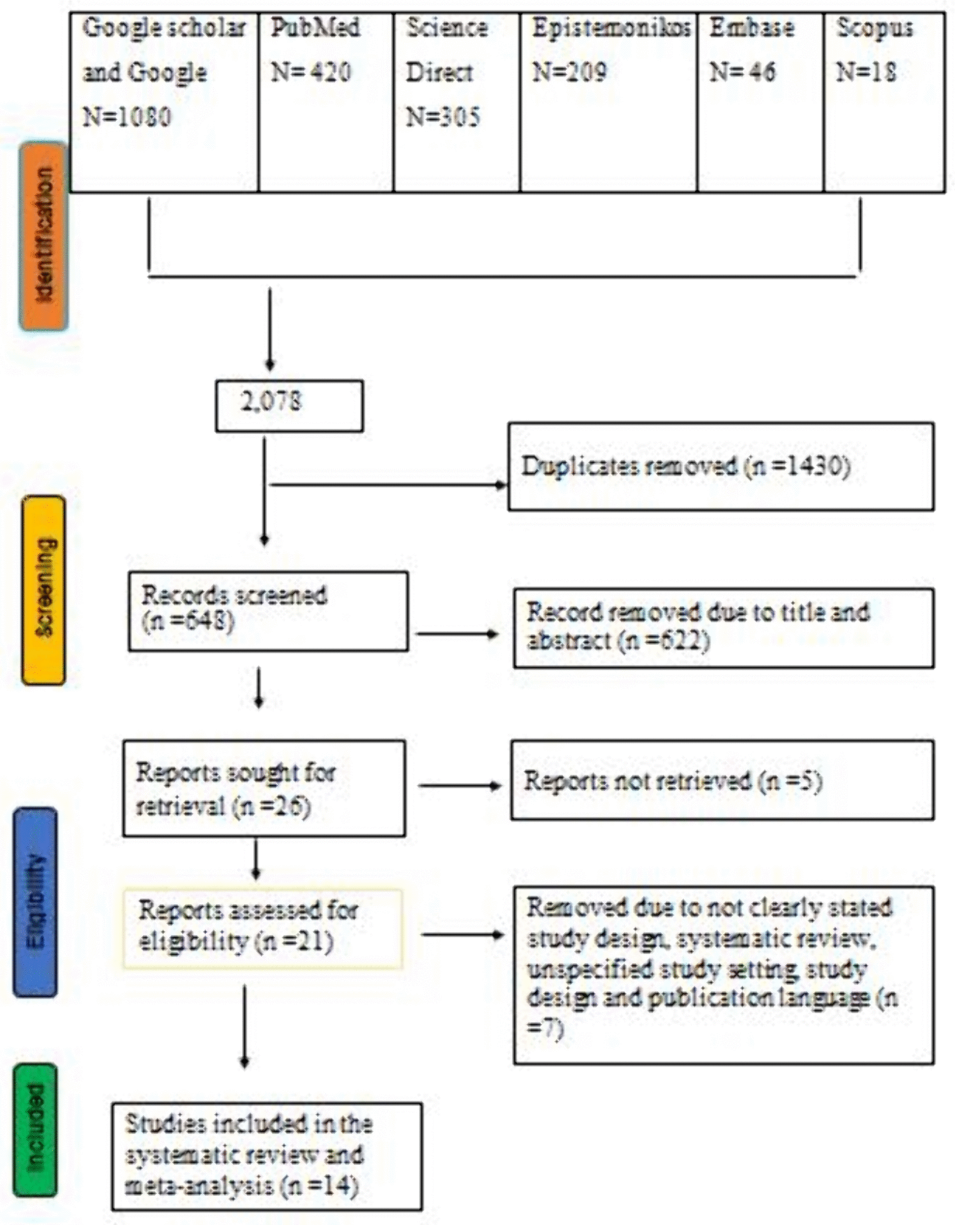
PRISMA 2020 flow diagram of study selection for the systematic review and meta-analysis on the global pooled prevalence of scabies among forcibly displaced populations, 2025.

A total of 2,078 articles were initially identified through searches of electronic databases, including PubMed, Epistemonikos, ScienceDirect, Embase, and Scopus, along with manual searches via Google and Google Scholar. After removing 1,430 duplicates using EndNote, 622 records were excluded during title and abstract screening. Five additional articles could not be retrieved, and seven were excluded after full-text assessment for reasons such as being systematic reviews, lack of full-text availability, unclear study setting, non-English publication, or unspecified study design. In the end, 14 studies fulfilled all eligibility criteria and were included in the final systematic review and meta-analysis (Fig 1).

### Characteristics of included studies

This systematic review and meta-analysis synthesized data from 14 studies, encompassing a total sample size of 76,664 individuals. The studies were geographically distributed across Asia (n = 5), Europe (n = 5), and Africa (n = 4), with Germany (n = 4) and Iraq (n = 3) contributing the highest number of studies. Regarding the study settings, six studies were conducted in internally displaced persons (IDP) camps, another six in refugee camps, one among asylum seekers, and one involving both refugees and asylum seekers. In terms of study populations, five studies assessed the general population, five focused on dermatology or skin disease patients, one examined unaccompanied minors, one targeted women and children, one focused on school-aged children, and one included both adults and children. Sample sizes ranged widely, from 374 participants in a study from Chad [29] to 33,410 participants in a study from Ethiopia [37]. Reported prevalence of scabies also varied considerably, with the lowest observed in Germany at 1.4% [28] and the highest in Bangladesh at 66.42% [23] (Table 2). The table summarizes study details, including Author name, year of publication, study location, country, study design, sample size, response rate, the number of cases, and prevalence of scabies.

**Table 2:** Characteristics of studies included in the systematic review and meta-analysis of scabies prevalence among forcibly displaced populations.

Author (s) and Publication Year	Study location and Country	Study design	Sample size	Response rate (%)	Cases (n)	Prevalence (%)
Lafta R., et.al, 2016 [27]	Kirkuk, Baghdad, and Karbala, Iraq	Cross sectional	4879	100	305	6.25
Theuring S., et.al, 2016 [28]	Berlin, Germany	Cross sectional	1248	100	17	1.4
Ismael AF and El-Gilany A. 2015 [29]	Southern Chad, Chad	Cross sectional	374	100	20	5.35
Rasul MM., et.al, 2019 [1]	Kaga Bandoro district, Central African Republic	Cross sectional	1561	100	74	7.22
Rahman MS., et.al, 2024 [23]	Teknaf/Ukhia Cox’s Bazar, Bangladesh	Cross sectional	679	100	450	66.42
Abdullah AM., et.al, 2020 [30]	Duhok Refugees’ Camps, Iraq	Cross sectional	6264	100	342	5.45
Zinszer K, and Abuzerr S., 2024 [31]	Gaza, Palestine	Cross sectional	1500	100	38	2.5
Kortas AZ., et.al, 2017 [32]	Germany	Cross sectional	2602	100	44	1.7
Wollina U., et.al, 2016 [33]	Dresden, Germany	Cross sectional	1100	100	16	1.45
Di Meco E., et.al, 2018 [34]	Lampedusa and Trapani-Milo, Italian	Cross sectional	6188	100	3589	58
Alberfkani MI, and Mero WM, 2020 [35]	Cham Mishko Camp, Zakho City, Iraq	Cross sectional	1300	100	585	45
Ibrahim AM., et.al, 2025 [36]	Qoloji IDP in Babile district, Somalia	Cross sectional	422	100	63	14.92
Alemu T., et.al, 2022 [37]	Ranch collective site, Chagni town, Ethiopia	Cross sectional	33410	100	1454	4.35
Alberer M., et.al, 2018 [38]	Munich, Germany	Cross sectional	15137	100	310	2.05

*Cross-sectional studies are observational studies that analyze data from a population at a single point in time of data collection.

### Meta-analysis

#### Global prevalence of scabies among forcibly displaced people.

**Fig 2:**
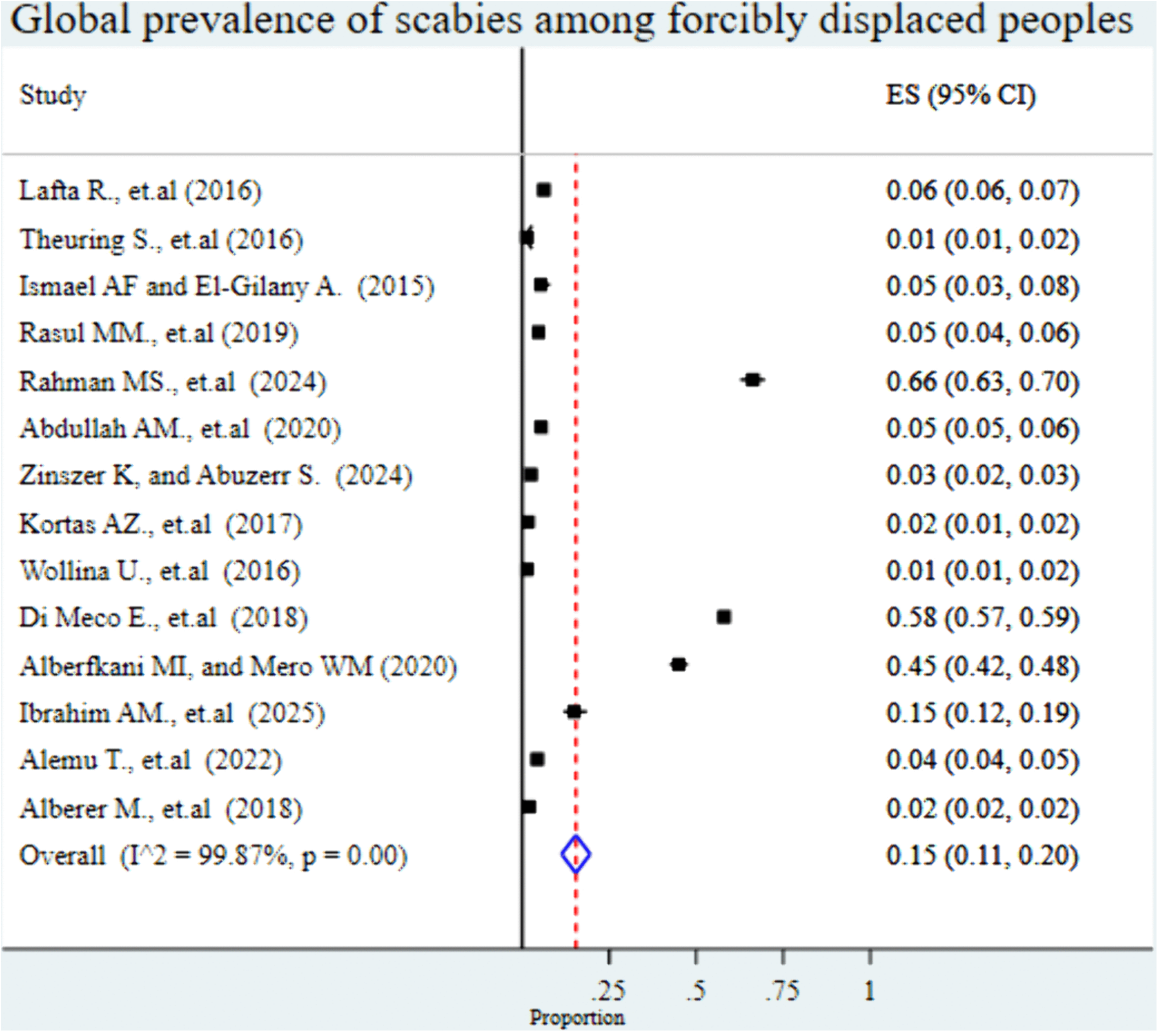
Forest plot showing the global pooled prevalence of scabies among forcibly displaced populations. This figure presents the overall prevalence estimate with 95% confidence intervals from the included 14 studies.

Across 14 studies, the global pooled prevalence of scabies among forcibly displaced populations was estimated at 15% (95% CI: 11%-20%). The highest prevalence was reported in a study from Bangladesh, at 66% (95% CI: 63%-70%) [23], followed by a study conducted in Italy with a prevalence of 58% (95% CI: 57%-59%) [34]. In contrast, the lowest prevalence was observed in Germany, at 1% (95% CI: 1%-2%) [28,33]. Substantial heterogeneity was detected among the included studies, as indicated by the I^2^ statistic (I^2^ = 99.87%, p < 0.001) (Fig 2). Given the extremely high heterogeneity, the pooled prevalence should be interpreted as an indicative summary measure rather than a definitive global estimate. To explore potential sources of heterogeneity, subgroup and sensitivity analyses were performed.

#### Galbraith plot and heterogeneity assessment.

**Fig 3:**
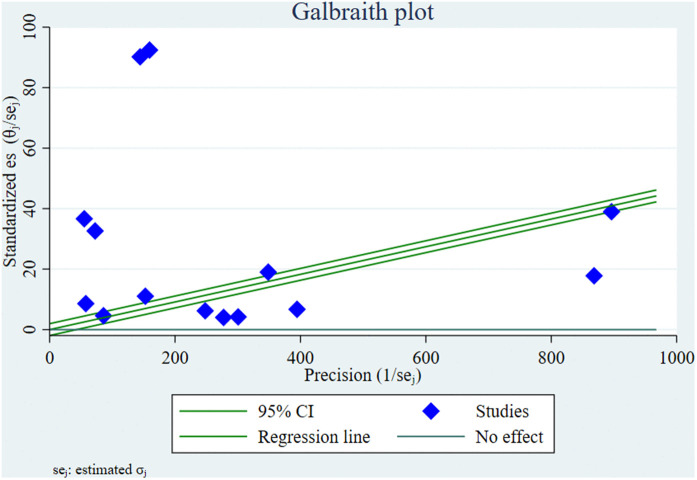
Galbraith (radial) plot of the included 14 studies illustrating the magnitude of between-study heterogeneity.

A Galbraith plot was generated to assess the heterogeneity among the included 14 studies in the meta-analysis. In this plot, each study is represented as a point, with the standardized effect size plotted against the inverse of its standard error. The majority of the studies fall outside the standard deviation lines, indicating substantial heterogeneity across the studies. The wide scattering of points and their deviation from the central regression line further confirm the inconsistency among study findings. This visual evidence supports the statistical findings of significant heterogeneity consistent with the I^2^ statistic. This dispersion indicates that the pooled estimate should be interpreted cautiously and justifies the use of a random-effects model and further subgroup or meta-regression analyses to explore potential sources of variability (Fig 3).

#### Subgroup analysis for the prevalence of scabies among forcibly displaced people.

To identify potential sources of heterogeneity, subgroup analyses were performed based on continent, subcontinent, study setting, study population, data collection method, and publication year.

**Fig 4:**
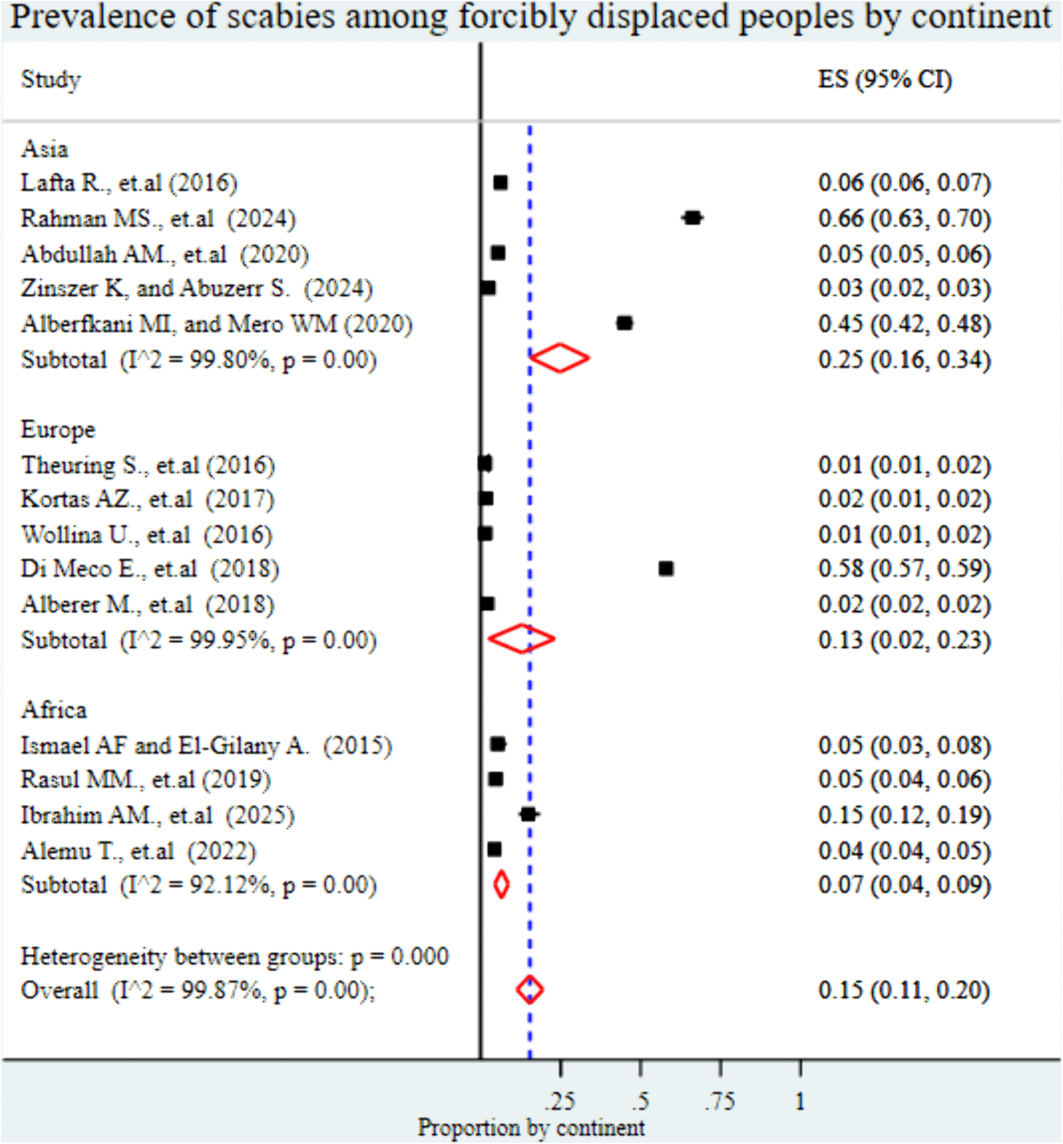
Forest plot showing the pooled prevalence of scabies among forcibly displaced populations, stratified by continent. This figure presents prevalence estimates from studies conducted in Asia, Africa, and Europe, highlighting regional variation.

**Fig 5:**
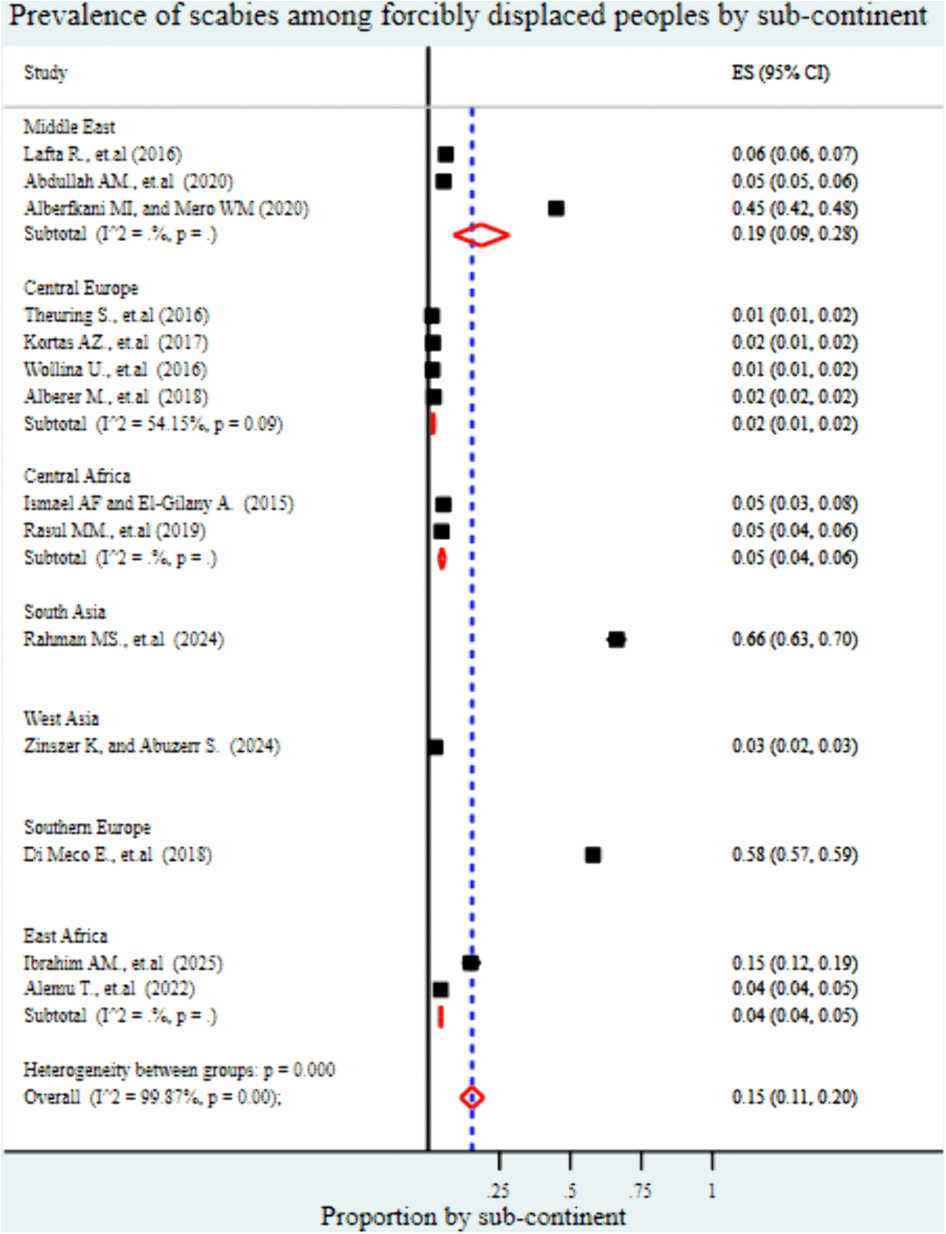
Forest plot showing the pooled prevalence of scabies among forcibly displaced populations, stratified by subcontinent. Prevalence estimates are shown for sub-regions including South Asia, Southern Europe, Central Europe, Central Africa, East Africa, West Asia, and the Middle East.

The analysis by continent showed that the highest pooled prevalence of scabies was reported in Asia at 25% (95% CI: 16%-34%), followed by Europe at 13% (95% CI: 2%-23%), and the lowest in Africa at 7% (95% CI: 4%-9%) (Fig 4). At the subcontinental level, the highest pooled prevalence of scabies was observed in South Asia at 66% (95% CI: 43%-70%), followed by Southern Europe at 58% (95% CI: 57%-59%). The Middle East reported a prevalence of 19% (95% CI: 9%-28%), while Central Europe showed a much lower prevalence of 2% (95% CI: 1%-2%). In Africa, prevalence estimates were 5% (95% CI: 4%-5%) in Central Africa, 4% (95% CI: 4%-5%) in East Africa, and 3% (95% CI: 2%-3%) in West Asia (Fig 5).

**Fig 6:**
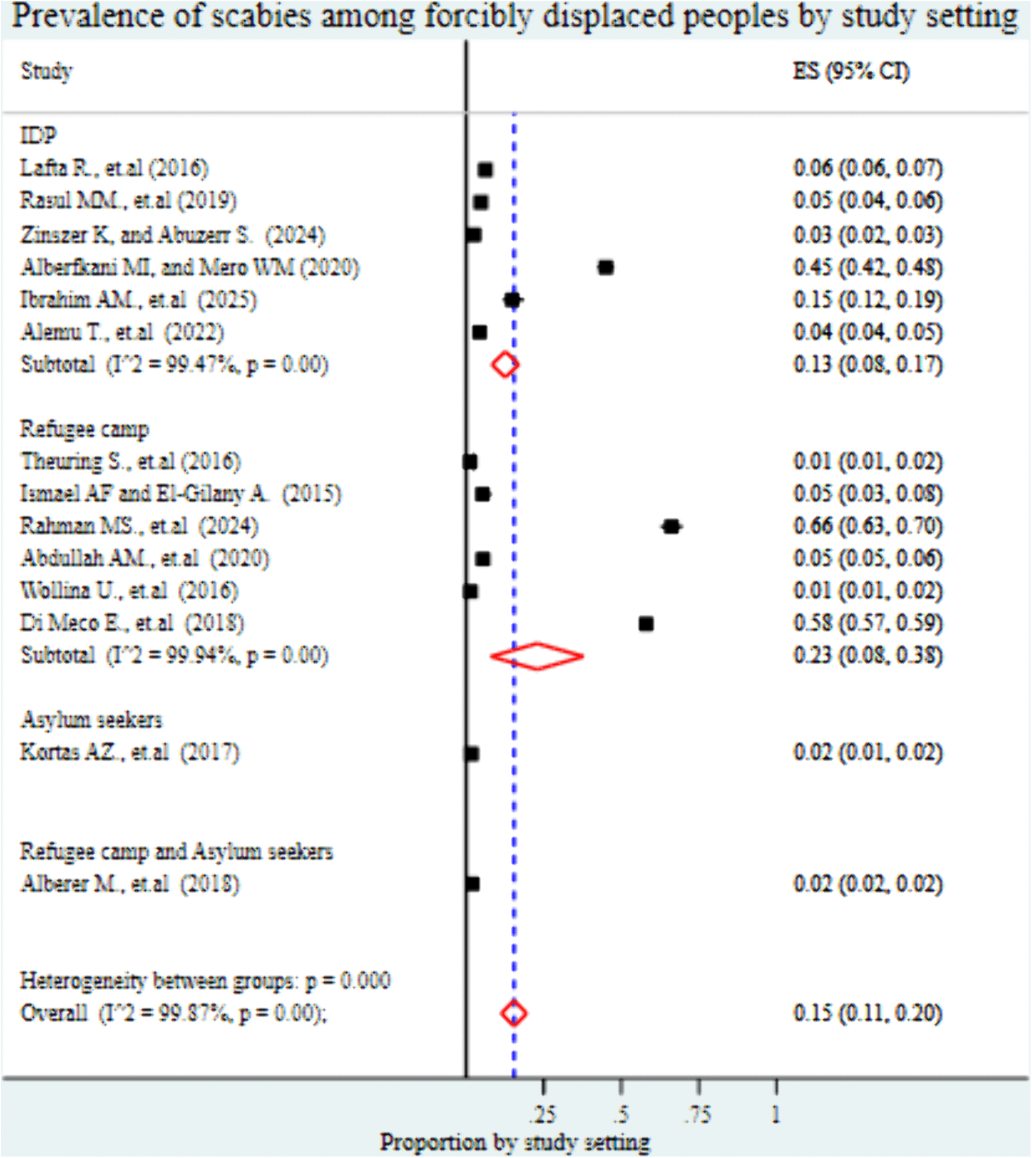
Forest plot showing the pooled prevalence of scabies among forcibly displaced populations, stratified by study setting. Settings include refugee camps, internally displaced persons (IDP) camps, asylum seeker centers, and studies involving both refugee and asylum-seeking populations.

**Fig 7:**
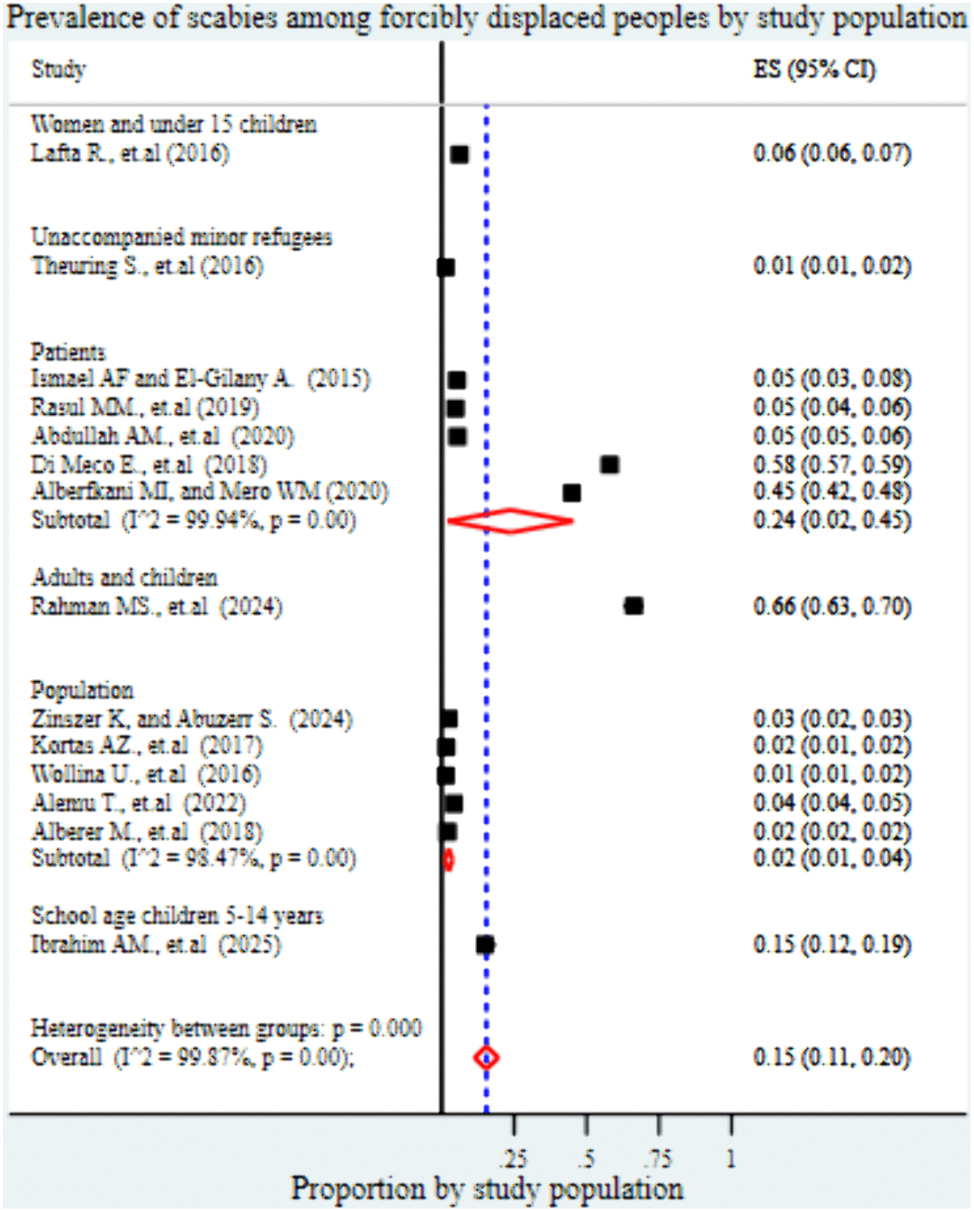
Forest plot showing the pooled prevalence of scabies among forcibly displaced populations, stratified by study population type. The analysis distinguishes between studies conducted among patient populations, women and children under 15, unaccompanied minor refugees, adults- children, and those conducted among the general forcibly displaced population.

Regarding study setting, the pooled prevalence of scabies was 13% (95% CI: 8%-17%) in internally displaced persons (IDP) camps, 23% (95% CI: 8%-38%) in refugee camps, 2% (95% CI: 1%-2%) among asylum seekers, and 2% (95% CI: 1%-2%) in studies involving both refugee camps and asylum seeker (Fig 6). In terms of study population characteristics, the highest prevalence was observed among combined adult and child populations, at 66% (95% CI: 63%-70%), followed by studies conducted specifically among patients, with a prevalence of 24% (95% CI: 2%-45%). School-aged children showed a pooled prevalence of 15% (95% CI: 12%-19%), while studies focusing on women and children under 15 years reported a prevalence of 6% (95% CI: 6%-7%). In contrast, studies conducted among the general population and unaccompanied minor refugees reported significantly lower prevalence rates, at 2% (95% CI: 1%-4%) and 1% (95% CI: 1%-2%), respectively (Fig 7).

**Fig 8:**
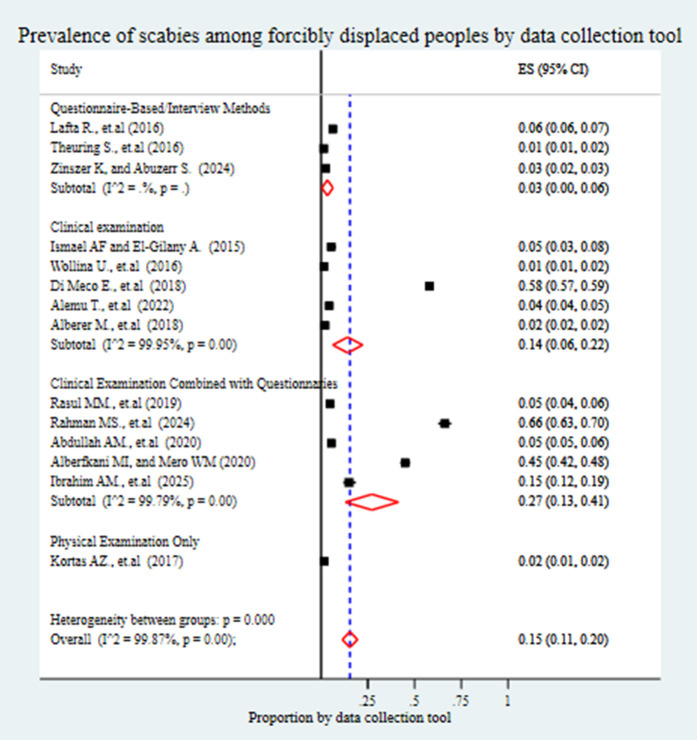
Forest plot showing the pooled prevalence of scabies among forcibly displaced populations, stratified by data collection method. The subgroup analysis compares studies using clinical examination only, clinical examination combined with Questionnaires, questionnaire/interview-based methods, and physical examination only.

**Fig 9:**
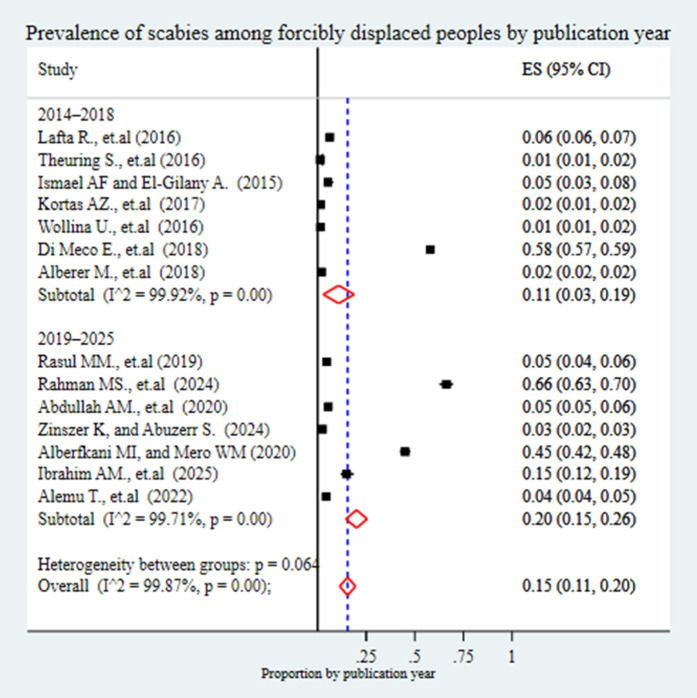
Forest plot showing the pooled prevalence of scabies among forcibly displaced populations, stratified by publication year. Studies are grouped into two publication periods: 2014–2018 and 2019–2025, to examine possible temporal trends in reported prevalence.

Based on the method of data collection, the pooled prevalence of scabies was 14% (95% CI: 6%-22%) in studies that utilized clinical examination alone. A higher prevalence of 27% (95% CI: 13%-41%) was observed in studies that combined clinical examination with Questionnaires. In contrast, studies that relied solely on Questionnaires or interviews reported a prevalence of 3% (95% CI: 0%-6%), while those using only physical examination showed the lowest prevalence at 2% (95% CI: 1%-2%) (Fig 8). When stratified by publication year, studies published between 2014 and 2018 reported a pooled prevalence of 11% (95% CI: 3%-19%), while those published between 2019 and 2025 showed a higher prevalence of 20% (95% CI: 15%-26%) (Fig 9).

Statistically significant heterogeneity was observed between subgroups based on continent, subcontinent, study setting, study population, and data collection method (p = 0.00), indicating that these factors contributed to the observed variation in prevalence estimates. However, subgroup analysis based on publication year did not show statistically significant heterogeneity (p = 0.064), although the borderline p-value suggests a potential temporal trend. In general, during these subgroup analyses, the I^2^ test showed there was still heterogeneity across the studies.

### Sensitivity analysis

**Fig 10:**
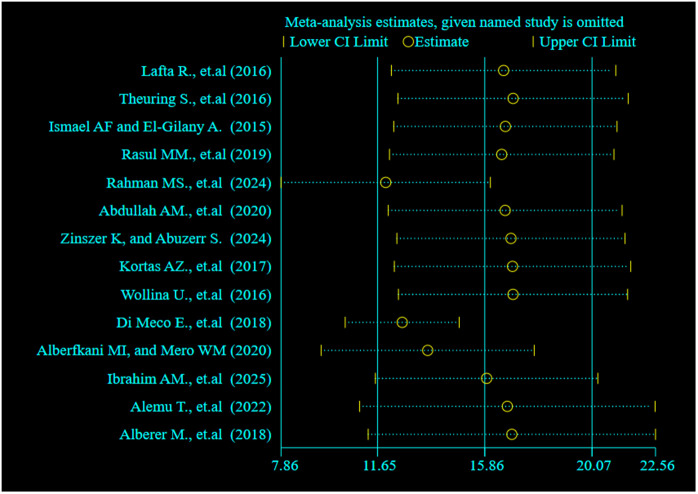
Sensitivity analysis plot assessing the influence of individual studies on the pooled prevalence of scabies among forcibly displaced populations. The figure illustrates the stability of the pooled estimate when each study is omitted sequentially from the meta-analysis.

A sensitivity analysis was conducted to assess the influence of individual studies on the overall pooled prevalence of scabies among forcibly displaced populations. This analysis involved sequentially excluding each of the 14 included studies to observe any significant changes in the overall estimate. The results showed that the pooled prevalence remained stable across all iterations, indicating that no single study had a disproportionate effect on the overall meta-analysis model. This suggests that the findings are robust and not unduly influenced by any one study (Fig 10). The consistency of the results also reinforces the reliability of the pooled estimate despite the presence of heterogeneity among studies.

### Small study effect test (Assessment of publication bias)

#### Assessment of publication bias.

**Fig 11:**
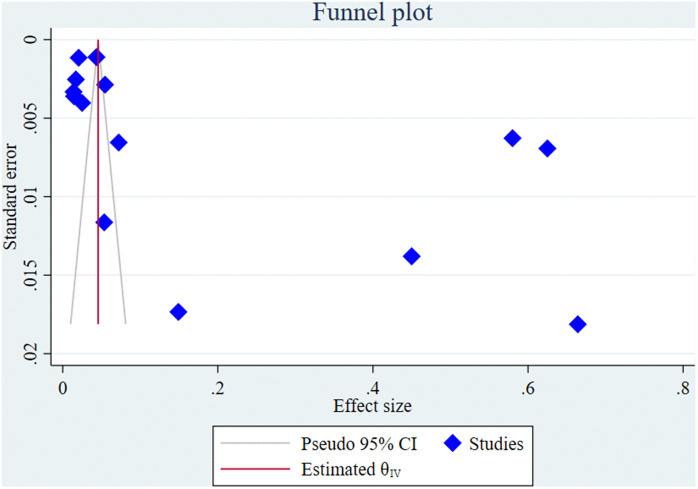
Funnel plot of the 14 studies included in the meta-analysis assessing the prevalence of scabies among forcibly displaced populations. The plot was used to visually assess potential publication bias (small study effects).

**Fig 12:**
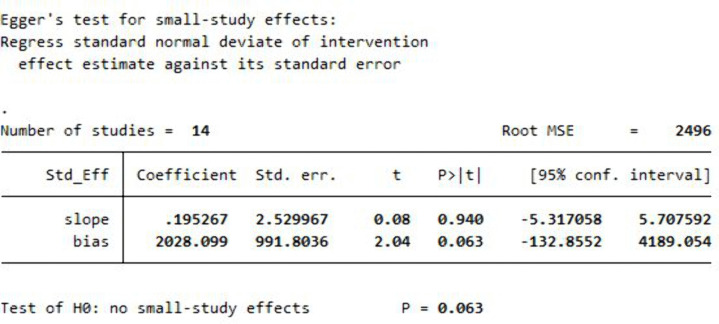
Egger’s test plot for the 14 studies included in the meta-analysis assessing the prevalence of scabies among forcibly displaced populations. The test was conducted to statistically evaluate the presence of publication bias (small study effects).

Publication bias or small study effect was evaluated using both visual inspection of a funnel plot and statistical testing via Egger’s regression test. The funnel plot (Fig 11) demonstrated a relatively symmetric distribution of effect sizes around the pooled estimate, suggesting the absence of publication bias. Furthermore, the result of Egger’s test did not indicate statistical evidence of small study effects, with a p-value of 0.063 (Fig 12). Although this p-value is slightly above the conventional threshold of significance (p < 0.05), it does not support the presence of significant publication bias. These findings collectively suggest that the meta-analysis results are not substantially influenced by bias from smaller studies or selective publication.

## Discussion

This study synthesized evidence from 14 relevant studies to estimate the pooled prevalence of scabies among forcibly displaced populations, including individuals residing in IDP camps, refugee settlements, and among asylum seekers across various continents. The combined sample comprised 76,664 participants. The findings revealed a considerable degree of variation in scabies prevalence across the included studies, underscoring the heterogeneity of the burden in different settings and population groups.

The pooled prevalence of scabies among forcibly displaced populations in this meta-analysis was estimated at 15% (95% CI: 11%–20%). This finding is consistent with previous systematic reviews and meta-analyses, such as a global review reporting a prevalence of 11.9% across all age groups [45], Ethiopia on all age groups (14.5%) [46], and another in Ethiopia focusing on school-aged children (14.71%) [47]. However, the prevalence reported in this study is lower than that found in a meta-analysis conducted in Ethiopia, specifically among school-aged children (21.1%) [48]. Conversely, the prevalence observed here is higher than estimates from other population groups, such as prisoners globally [49], school-aged children in Africa (10.81%) [50], and prisoners in southern Ethiopia (8.9%) [51]. These variations may be attributed to several factors, including differences in study populations, geographic and environmental contexts, study years, as well as behavioral and cultural practices. The forcibly displaced populations often reside in overcrowded environments with poor sanitation, limited access to water, and inadequate hygiene facilities. Such conditions facilitate close skin-to-skin contact and reduce the capacity for effective disease prevention and control, thereby contributing to a higher burden of scabies in these settings. Moreover, disparities in socioeconomic status, health infrastructure, and health-seeking behavior among forcibly displaced communities may exacerbate scabies transmission. These challenges are often more pronounced in displacement settings than among general or institutionalized populations, such as prisoners or schoolchildren in stable communities. Hence, the elevated prevalence in forcibly displaced populations may reflect a combination of structural, environmental, and behavioral vulnerabilities unique to humanitarian crises.

To assess the presence and degree of heterogeneity among the included studies, a Galbraith plot was generated for visualization, and the I^2^ statistic revealed substantial heterogeneity (I^2^ = 99.87%, *p* < 0.001). This high level of heterogeneity suggests that the observed variability in scabies prevalence estimates is unlikely to be due to chance alone [52], warranting further investigation into potential sources of this variation. Consequently, subgroup analyses were performed based on continent, subcontinent, study setting, study population, year of publication, and data collection method. Statistically significant differences in scabies prevalence were observed across subgroups defined by continent, subcontinent, study setting (e.g., IDP vs. refugee camps), study population characteristics, and data collection methods. These findings suggest that geographic, demographic, and methodological factors may substantially influence the reported burden of scabies in forcibly displaced populations. Such an approach aligns with best practices in meta-analysis for identifying and addressing heterogeneity in pooled estimates [53].

The subgroup analysis demonstrated notable regional variation in the pooled prevalence of scabies among forcibly displaced populations. The highest prevalence was observed in Asia at 25% (95% CI: 16%–34%), followed by Europe at 13% (95% CI: 2%–23%), with the lowest prevalence recorded in Africa at 7% (95% CI: 4%–9%). These differences may be attributed to a combination of environmental and structural factors common to displacement settings. Overcrowding, inadequate sanitation and hygiene (WASH) conditions, and malnutrition all contribute to the spread of disease [54–58]. Such conditions are highly conducive to the transmission of scabies, which spreads primarily through prolonged skin contact and shared living spaces. Around 2.4 billion people globally lack access to basic sanitation, and the majority of these unserved people (93%) live in Asia and Africa [59]. These regions host large numbers of forcibly displaced populations living in temporary or under-resourced settlements [60]. Therefore, the higher prevalence of scabies in certain regions may reflect broader systemic deficiencies in humanitarian settings, emphasizing the urgent need for improved WASH interventions and targeted public health measures in displacement contexts.

The subgroup analysis revealed significant differences in the pooled prevalence of scabies across various displacement settings. The highest prevalence was recorded in refugee camps, at 23% (95% CI: 8%-38%), followed by IDP camps at 13% (95% CI: 8%-17%). In contrast, a much lower prevalence was observed among asylum seekers (2%, 95% CI: 1%-2%) and in studies involving both refugee and asylum seeker populations (2%, 95% CI: 1%-2%). These disparities may be explained by differences in living conditions, population density, and access to healthcare services across settings. Refugee and IDP camps are often overcrowded, under-resourced, and face significant challenges in maintaining basic WASH infrastructure, which facilitates the transmission of scabies [61]. In many of these camps, individuals may share bedding, clothing, or have prolonged skin-to-skin contact conditions that are ideal for mite transmission. In contrast, asylum seekers, particularly those housed in higher-income countries or temporary reception facilities, may benefit from better living standards, access to hygiene, and early health screenings, which can reduce the spread of disease [62]. Refugee and IDP populations may face barriers to accessing medical care, including logistical constraints, understaffed health posts, language difficulties, and fear of discrimination or deportation which leads to delayed diagnosis and treatment [63,64], while asylum seekers may undergo routine medical screening as part of resettlement procedures, which facilitates early detection and management of infectious diseases, including scabies [65].

The subgroup analysis based on study population characteristics revealed substantial variation in the pooled prevalence of scabies. The highest prevalence was observed among combined adult and child populations, at 66% (95% CI: 63%-70%), followed by studies conducted specifically among patients, with a prevalence of 24% (95% CI: 2%-45%). School-aged children showed a pooled prevalence of 15% (95% CI: 12%-19%), while studies focusing on women and children under 15 years reported a prevalence of 6% (95% CI: 6%-7%). In contrast, studies conducted among the general population and unaccompanied minor refugees reported significantly lower prevalence rates, at 2% (95% CI: 1%-4%) and 1% (95% CI: 1%-2%), respectively. This variation may reflect differences in vulnerability and exposure risks among subgroups. Higher prevalence rates among adult–child and clinical patient populations may be due to more severe presentations that lead to healthcare seeking and thus higher detection rates [61,66]. Children, especially those of school age, are particularly vulnerable to scabies due to close contact in communal settings, such as classrooms and shared sleeping areas. Studies have shown that scabies disproportionately affects children in overcrowded or resource-limited settings [48,50]. These findings highlight the importance of tailoring public health interventions and surveillance systems according to population subgroups, with a particular focus on children and populations in overcrowded settings where the risk of transmission is highest.

Furthermore, this study identified substantial variation in the reported prevalence of scabies depending on the method of data collection. The pooled prevalence was 14% (95% CI: 6%-22%) in studies using clinical examination alone, and markedly higher at 27% (95% CI: 13%-41%) in studies that combined clinical examination with Questionnaires. In contrast, studies relying solely on questionnaire- or interview-based assessments reported a lower pooled prevalence of 3% (95% CI: 0%-6%), while those using physical examination only showed the lowest prevalence, at 2% (95% CI: 1%-2%). These differences may be attributed to the varying sensitivity and specificity of diagnostic methods. Studies that incorporated both clinical examination and self-reported symptoms via Questionnaires likely captured more cases, including mild or subclinical presentations, resulting in a higher prevalence estimate. In contrast, questionnaire-only approaches may lead to underestimation due to recall bias, stigma-related underreporting, or lack of awareness of scabies symptoms, especially in low-literacy or resource-poor settings [13,67]. Additionally, the accuracy of diagnosis through physical examination alone may be limited by the examiner’s experience and the nonspecific nature of scabies lesions, which often resemble other dermatological conditions. Consequently, variations in data collection methods directly influence the reliability and comparability of prevalence estimates, highlighting the need for standardized and validated diagnostic protocols in scabies epidemiology [66].

## Strengths and limitations of the study

This systematic review and meta-analysis offer important insights into the global pooled prevalence of scabies among forcibly displaced populations, providing a consolidated understanding of the burden of disease in these vulnerable groups. A major strength of this study lies in its comprehensive approach, drawing on data from diverse displacement settings and applying rigorous meta-analytic techniques to quantify prevalence.

However, several limitations should be acknowledged. First, the review was restricted to studies published in English, which may have excluded relevant data published in other languages and introduced language bias, thereby limiting the completeness of the evidence base. Second, all included studies were observational in design, which increases the risk of residual confounding and limits the ability to infer causality. Third, although this review aimed for global representation, eligible studies were only available from three continents. As a result, the geographic distribution of included data may limit the broader generalizability of the findings. Future research should aim to address this gap by incorporating non-English language studies, expanding the geographic coverage, and generating high-quality, regionally representative data from underreported areas, where forcibly displaced populations also reside. Such efforts would contribute to a more comprehensive and globally representative understanding of scabies prevalence among forcibly displaced populations.

## Conclusions and recommendations

This systematic review and meta-analysis, which synthesized data from 14 studies, estimated the global pooled prevalence of scabies among forcibly displaced populations to be 15%. However, given the extremely high heterogeneity (I^2^ = 99.87%), this estimate should be interpreted with caution and viewed as an indicative summary measure rather than a precise global burden. Considerable variation was observed across geographic regions, study settings, population groups, and diagnostic approaches. The highest pooled prevalence was reported in Asia (25%), followed by Europe (13%) and Africa (7%). In terms of study setting, scabies prevalence was highest among refugees (23%), followed by IDPs (13%), and substantially lower among asylum seekers and mixed refugee–asylum populations (2%) each. Population-specific analyses revealed the highest prevalence among the combined adult–child study (66%), followed by patients (24%) and school-aged children (15%). Lower prevalence rates were observed among women and children under 15 years (6%), the general population (2%), and unaccompanied minor refugees (1%). Furthermore, differences in prevalence were also influenced by data collection methods: studies using both clinical examinations and Questionnaires yielded the highest prevalence (27%), 14% in studies using clinical examination alone, while those relying solely on interviews or physical examination reported significantly lower estimates (3% and 2%, respectively).

These findings highlight the considerable burden of scabies among forcibly displaced populations and point to critical disparities across geographic regions, population groups, and diagnostic approaches. Tailored interventions are essential to address these differences. Public health efforts should prioritize regular scabies screening, prompt diagnosis, and mass drug administration (MDA) campaigns in high-prevalence settings, particularly in refugee and IDP camps where overcrowding and poor sanitation are prevalent. Additionally, integrating scabies control with water, sanitation, and hygiene (WASH) programs and health education initiatives can significantly reduce transmission. Strengthening surveillance systems and improving diagnostic capacity in low-resource, high-risk settings should be prioritized. From a policy perspective, coordinated international support and long-term investment in scabies prevention strategies are critical to protect vulnerable displaced communities and reduce the risk of outbreaks.

## Supporting information

S1 File PRISMA 2020 Checklist - *Both documents are distributed under the terms of the Creative Commons Attribution License (CC BY 4.0), which permits unrestricted use, distribution, and reproduction in any medium or format for any purpose, provided the original author and source are credited.* (DOCX)

S2 File Searching strategies and documentation for the burden of forcibly displaced populations. (DOCX)

S3 File Newcastle-Ottawa Scale (NOS) Quality assessment of papers. (DOCX)

S4 File The extracted Excel file for the burden of forcibly displaced populations. (XLSX)
