# Supplementary material for: Burden of scabies in displacement settings: A systematic review and meta-analysis among forcibly displaced populations
Source: PLoS Negl Trop Dis. 2025 Dec 23;19(12):e0013853. doi: 10.1371/journal.pntd.0013853 (PMC12725569; doi:10.1371/journal.pntd.0013853)
Supplement: S2 File — (DOCX) [file pntd.0013853.s002.docx]

**Searching document for prevalence of scabies among forcibly displaced peoples peoples**

| Mesh Heading | Entry words | Data base | combination | Number of studies | Searching date |
| --- | --- | --- | --- | --- | --- |
| Scabies | - scabies | PubMed | (((((((prevalence of scabies[Title/Abstract]) OR (magnitude of scabies[Title/Abstract])) AND (internally displaced people[Title/Abstract])) OR (refugee camp people[Title/Abstract])) OR (forcibly displaced people[Title/Abstract])) OR (migrant camp[Title/Abstract])) OR (armed conflict people[Title/Abstract])) OR (forcibly migrant people[Title/Abstract]) | 420 | Wednesday May 28, 2025 |
| Forcibly displaced people | - Refugee camp people - Internally displaced people - Asylum seeking peoples - Migrated peoples - Migration camps - forcibly displaced people - conflict affected people - forced migration people - armed conflict affected people | Google scholar | Prevalence of scabies among refugee camp and Internally displaced people "scabies" AND "internally displaced people *" OR "refugee camp people *" OR "forcibly displaced people *" OR "conflict affected people *" OR "forced migration people *" OR "armed conflict affected people *" "scabies" | 1080 | Tuesday, May 27, 2025 |
|  |  | SCOPUS | (title:(scabies AND internally displaced people) OR abstract:( scabies AND internally displaced people)) OR (title:(diarrhea AND refugee camp people) OR abstract:( scabies AND refugee camp people)) OR (title:( scabies AND forcibly displaced people) OR abstract:( scabies AND forcibly displaced people)) OR (title:( scabies AND conflict affected people) OR abstract:( scabies AND conflict affected people)) | 18 | Tuesday, May 27, 2025 |
|  |  | EMBASE | ('scabies'/exp OR scabies) AND 'internally displaced people' OR 'refugee camp people' OR 'forcibly displaced people' OR 'conflict affected people' OR 'migrated people' | 46 | Wednesday May 28, 2025 |
|  |  | COCHRAN | "prevalence of scabies" AND "internally displaced people" OR "refugee camp people" OR “forcibly displaced people” OR "conflict affected people " OR "forcibly migrated people" OR "armed conflict affected people" OR "migrant camp" in Title Abstract Keyword | 0 | Wednesday May 28, 2025 |
|  |  | ScienceDirect | "prevalence” of “scabies" AND "internally displaced people" OR "refugee camp people" OR “forcibly displaced people” OR "conflict affected people " OR "forcibly migrated people" OR "armed conflict affected people" OR "migrant camp" | 305 | Wednesday May 28, 2025 |
|  |  | Epistemonikos | (advanced_title_en:(prevalence of scabies) OR advanced_abstract_en:(prevalence of scabies)) AND (advanced_title_en:(internally displaced people*) OR advanced_abstract_en:(internally displaced people*)) OR (advanced_title_en:(refugee camp people*) OR advanced_abstract_en:(refugee camp people*)) OR (advanced_title_en:(migrant camp people*) OR advanced_abstract_en:(migrant camp people*)) OR (advanced_title_en:(forcibly displaced people*) OR advanced_abstract_en:(forcibly displaced people*)) OR (advanced_title_en:(conflict affected people*) OR advanced_abstract_en:(conflict affected people*)) OR (advanced_title_en:(forced migration people) OR advanced_abstract_en:(forced migration people)) OR (advanced_title_en:(armed conflict affected people*) OR advanced_abstract_en:(armed conflict affected people*)) | 209 | Wednesday May 28, 2025 |
| Total paper |  | From all 7 data base |  | 2,078 |  |
