# Supplementary material for: Burden of scabies in displacement settings: A systematic review and meta-analysis among forcibly displaced populations
Source: PLoS Negl Trop Dis. 2025 Dec 23;19(12):e0013853. doi: 10.1371/journal.pntd.0013853 (PMC12725569; doi:10.1371/journal.pntd.0013853)
Supplement: S3 File — (DOCX) [file pntd.0013853.s003.docx]

**Annex II: NOS quality assessment score of cross-sectional studies**

| **Authors, publication year and refrences** | **Study design** | **Selection** | | | | **Comparability** | **Outcome** | | **NOS quality** |
| --- | --- | --- | --- | --- | --- | --- | --- | --- | --- |
|  |  | **1** | **2** | **3** | **4** | **1** | **1** | **2** | **3** |
| Lafta R., et.al, 2016 [[36](#_ENREF_36)] | CS | A* | A* | A* | A** | B | C* | A* | Good |
| Theuring S., et.al, 2016 [[35](#_ENREF_35)] | CS | A* | A* | A* | A** | B | C* | A* | Good |
| Ismael AF and El-Gilany A. 2015 [[33](#_ENREF_33)] | CS | A* | A* | A* | A** | B | C* | A* | Good |
| Rasul MM., et.al, 2019 [[1](#_ENREF_1)] | CS | A* | A* | A* | A** | B | C* | A* | Good |
| Rahman MS., et.al, 2024 [[23](#_ENREF_23)] | CS | B* | A* | A* | A** | A** | B** | A* | Very Good |
| Abdullah AM., et.al, 2020 [[37](#_ENREF_37)] | CS | B* | A* | A* | A** | B | C* | A* | Good |
| Zinszer K, and Abuzerr S., 2024 [[38](#_ENREF_38)] | CS | A* | A* | A* | A** | A** | B** | A* | Very Good |
| Kortas AZ., et.al, 2017 [[39](#_ENREF_39)] | CS | A* | A* | A* | A** | B | C* | A* | Good |
| Wollina U., et.al, 2016 [[40](#_ENREF_40)] | CS | A* | A* | A* | A** | B | C* | A* | Good |
| Di Meco E., et.al, 2018 [[41](#_ENREF_41)] | CS | B* | A* | A* | A** | B | C* | A* | Good |
| Alberfkani MI, and Mero WM, 2020 [[42](#_ENREF_42)] | CS | B* | A* | A* | A** | A** | C* | A* | Very Good |
| Ibrahim AM., et.al, 2025 [[43](#_ENREF_43)] | CS | A* | A* | A* | A** | A** | B** | A* | Very Good |
| Alemu T., et.al, 2022 [[34](#_ENREF_34)] | CS | A* | A* | A* | A** | A** | C* | A* | Very good |
| Alberer M., et.al, 2018 [[44](#_ENREF_44)] | CS | A* | A* | A* | A** | B | C* | A* | Good |

CS: cross sectional study design

- **Selection: (maximum 5 stars)**

**1. Representativeness of the sample:**

a. Truly representative of the average in the target population. * (all subjects or random sampling)

b. Somewhat representative of the average in the target group. * (non-random sampling)

c. Selected group of users/convenience sample.

d. No description of the derivation of the included subjects.

**2. Sample size:**

a. Justified and satisfactory (including sample size calculation). *

b. Not justified.

c. No information provided

**3. Non-respondents:**

a. Proportion of target sample recruited attains pre-specified target or basic summary of non-respondent characteristics in sampling frame recorded. *

b. Unsatisfactory recruitment rate, no summary data on non-respondents.

c. No information provided

**4. Ascertainment of the exposure (risk factor):**

a. Vaccine records/vaccine registry/clinic registers/hospital records only. **

b. Parental or personal recall and vaccine/hospital records. *

c. Parental/personal recall only.

- **Comparability** **(Maximum 2 stars)**

1. Comparability of subjects in different outcome groups on the basis of design or analysis. Confounding factors controlled.

a. Data/ results adjusted for relevant predictors/risk factors/confounders e.g.

age, sex, time since vaccination, etc. **

b. Data/results not adjusted for all relevant confounders/risk factors/information not provided.

- **Outcomes (Maximum 3 stars)**

1**. Assessment of outcome:**

a. Independent blind assessment using objective validated laboratory methods. **

b. Unblinded assessment using objective validated laboratory methods. **

c. Used non-standard or non-validated laboratory methods with gold standard. *

d. No description/non-standard laboratory methods used.

**2. Statistical test:**

a. Statistical test used to analyses the data clearly described, appropriate and measures of association presented including confidence intervals and probability level (p value). *

b. Statistical test not appropriate, not described or incomplete

**Newcastle-Ottawa scale adapted for Cross-sectional studies**

1. Very good studies 9-10 points of star.
2. Good studies 7-8 points of star.
3. Satisfactory studies 5-6 points of star.
4. Unsatisfactory studies 0-4 points of star.
